# Analyses of actions which cause anterior cruciate ligament injuries in the national basketball association players: YouTube-based video analyses

**DOI:** 10.1186/s13102-023-00747-8

**Published:** 2023-10-12

**Authors:** Takanobu Saito, Natsuki Matsumura, Shinichi Kawamoto, Naoki Doi, Tomoki Aoyama, Momoko Nagai-Tanima

**Affiliations:** Human Health Sciences, Graduate School of Medicine, Kyoto University, 53 kawahara-cho, shogoin, Sakyo-ku, 606-8507 Kyoto, Japan

**Keywords:** Video analysis, Basketball, The National Basketball Association, Anterior cruciate ligament, Indirect contact

## Abstract

**Background:**

Anterior cruciate ligament (ACL) injuries are among the most common injuries in the National Basketball Association (NBA), and it is important to investigate the actual nature of the injury because it can impair a player’s performance after returning to the game. Although the moment of injury has been investigated, the details of the movements and circumstances leading to injury in basketball games are unknown. This study aimed to clarify the actions leading to ACL injuries and to investigate their characteristics, based on YouTube video analyses of the NBA players.

**Methods:**

Players with ACL injuries in the NBA were identified through web-based research over 10 seasons (2011/2012–2021/2022, through October 2021), with 29 recorded videos of ACL injuries in the NBA. Actions were categorized based on basketball-specific gestures, and determined whether the player was in contact with an opponent or not and, if so, the location of the contact was analyzed focusing on two time points: at the injury frame (IF) and one step before the injury frame (IF-1). The “injury leg” timing was counted for each of the first and second steps after ball possession.

**Results:**

The majority (68.2%) of ACL injury occurred during the 2 steps phase (only two steps can proceed after ball retention in basketball, so we defined them as two steps) in the offense action, and most notably during the first step (80.0%). 73.3% of players who were injured during the 2 steps phase got contact to an area other than the knee (Indirect contact) at the IF-1, with 81.8% of contact being located in the upper body contralateral to the respective knee injury. The probability of players with ACL injuries during the 2 steps at the IF-1 who got Indirect contact was statistically significantly greater than those who got no contact with other players (p = 0.042).

**Conclusions:**

We argue that including pre-injury play and contact falls into the novelty category. Through YouTube-based video analyses, this study revealed that ACL injuries tend to be characterized by specific types of actions, the timing of contact, and the location of contact in NBA players.

## Background

Basketball is a contact team sport, and the game develops quickly. In the National Basketball Association (NBA), a professional basketball league, injuries to players’ lower extremities are exceedingly common; in particular, knee injuries cause more missed games than any other injury to the body [1]. In fact, anterior cruciate ligament (ACL) injuries in the NBA are listed as one of the most common injuries based on their mean annual incidence, with an average incidence of 1.5–2.6% of total injuries of NBA per year; thus, it is considered among the most serious injuries to basketball players [2].

Compared to other professional leagues in other sports, the time between injury and recovery is longer in the NBA, moreover, there is a decline in performance in the season immediately following the return to play [3–6]. Therefore, to optimize the performance of NBA players, it is important to prevent ACL injuries.

Understanding the characteristics of situations and actions leading to injuries is crucial in preventing their occurrence [7]. Based on changes in the joint angle and ground force, the injury frame (IF) of ACL injury occurs 40 milliseconds (ms) after the initial contact [8]. While there are scattered studies that have examined the type of maneuver (e.g., cutting, landing, etc.) performed by the athlete at the moment of ACL injury, it is also helpful to analyze the type and characteristics of the movement leading to the maneuver to understand better the mechanism of ACL injury [8–10]. However, Krosshaug et al. only categorized the types of maneuvers in basketball as cutting, landing, and stopping, and few studies have investigated the playing action that leads to the point of injury [9, 11, 12]. Furthermore, it is important to consider the actions, situations, and contacts that occur not only at the IF but also before the injury, as conditions before the injury are thought to influence ACL injuries [12–14]. It is essential to consider not only the time of IF but also the actions, situations, and contact that occur before the injury, and there have been studies analyzing the pre-injury period in football separated by time, from 12 to 15 s before injury [12–15]. However, basketball game develops quickly, and the playing court is small. The impact of a single step is significant, so it is considered more practical to separate the period by play action rather than time, but there are no studies separated by play action.

Some study said ACL injuries could often be caused by contact to an area other than the knee (Indirect contact) by nearby opponents in NBA players on most occasions where only contact with the injured leg (Direct contact) or no contact with other players (Non-contact) at all is suggested [9, 14, 16]. Although ACL injury rates are high in the NBA and result in poor performance, and there are studies on the moment of injury, few have examined the sequence of events or considered contact with other parts of the body other than Direct contact.

Video analysis tools provide valuable information about the injury situation and are widely used to investigate behavior before and during the injury [17, 18]. As these video analyses have advanced our understanding of the characteristics of ACL injuries, in the current study, YouTube.com was used because of its easy access and necessary video resources [11].

Therefore, this study aimed to clarify the actions leading to ACL injuries and to investigate the characteristics of these actions, based on a YouTube video analysis of NBA players. This study hypothesizes that there may be a trend in the sequence of play leading to an ACL injury in NBA players; it may be possible to classify NBA players by the presence or absence of contact and the location of communication before and during ACL injury.

## Methods

### Search methods

Players in the NBA who suffered ACL injuries were identified through web research across 10 seasons (from the 2011/2012 season to 2021/2022 season, until October 2021) [11, 15]. These players were identified through NBA team websites, publicly accessible internet injury reports, player profiles, biographies, and November 2021 press releases [5]. This search allowed us to catch up on ACL injuries for all 10 seasons, and when an ACL lesion was confirmed, the injured player’s name was used to perform a search on YouTube [11]. In this study, videos uploaded by individuals based on factual information were used for noncommercial educational purposes. As such, it applies to the four elements of YouTube’s “fair use” and does not violate any policies or copyrights, and therefore does not require separate consent from the individual accounts that appear in the videos.

### Video processing

Every video included in the present analysis was downloaded and edited using Adobe Premiere (Adobe Systems Inc., San Jose, California) to allow for video repetitions, slow motion, and frame-to-frame navigation. Adobe Photoshop (Adobe Systems Inc., San Jose, California) was used to cut images from the videos [19]. All parameters were discussed between the two reviewers who were physical therapists until an agreement was reached in real time and in slow motion [11, 20].

### Evaluated parameters and video analysis

**Fig. 1:**
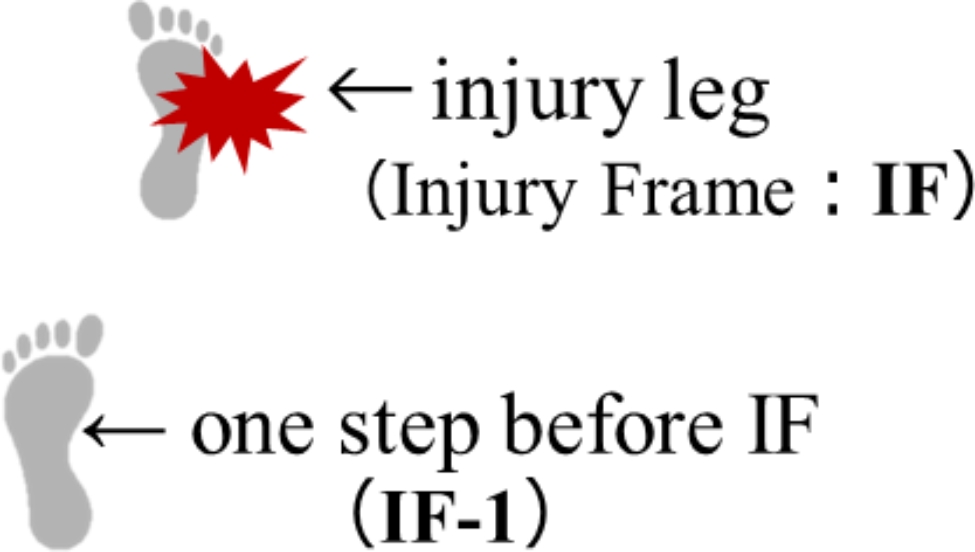
Injury leg (IF) and one step before IF (IF-1)

Demographic data (day of injury and age at the time of injury), information concerning position (guard, forward, center), injured leg (right, left), quarter of play (first, second, third, fourth) during which the injury occurred, NBA history at the time of injury, and ACL medical history were all collected via web research [5, 11]. In the present study, the step before IF was considered the pre-injury criterion because of the rapid gameplay changes. The IF was defined as 40ms after the initial contact of the injured leg, refer to the 40 ms after the moment when the injured leg touches the ground, with a consensus being established by the reviewers [8]. Therefore, each video of an ACL injury was identified as the IF and one step before the injury frame (IF-1) from the IF to evaluate the playing actions that preceded the injury and the mechanism underlying it [15] (Fig. 1).

**Fig. 2:**
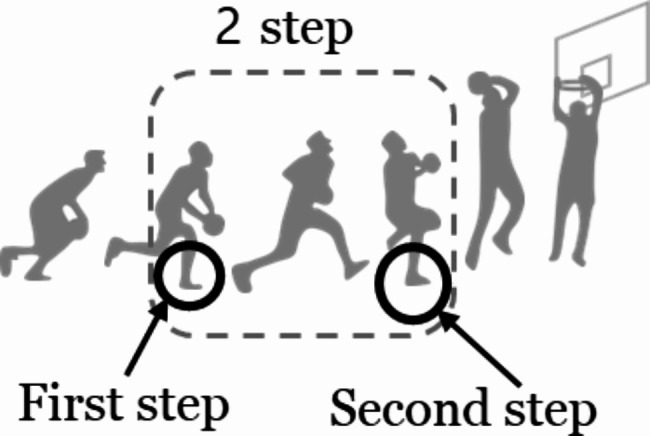
First step and second step in the sequence of shooting

Playing phase immediately before IF was classified into offense, defense, and rebound phases [9]. The offensive action immediately before and at the IF was related to the basketball-specific gesture performed by the injured player. The series of actions from the time the player grabs the ball until they land after shooting was defined as follows: dribble was defined as an action with intermittent touching of the ball with the continuous aim of going forward and avoiding opponents; 2 steps was defined as the action of holding the ball with the aim of shooting, stopping, or pivoting; and landing was defined as the action of landing after jumping for the purpose of shooting. For the 2 steps, we further classified whether the leg was injured during the first or second step (Fig. 2).

The position of the players in the field at the time of injury was accurately assessed by visual reference to the field lines, with it being plotted and reported on a scaled drawing equivalent to a basketball half-court [11]. The timing of contact was evaluated and classified into two types: the time span from IF-1 to immediately before the IF (at the IF-1) and at the IF from the same time series of the players. Contact was classified into three types: Direct contact, Indirect contact, and Non-contact. As the knee joint consists of the femur, tibia, and patella, we have defined contact with the femur, patella, and tibia on the injured side as “Direct contact.“ Contact with other body parts was described as “Indirect contact”. Indirect contact was further classified into four types, depending on the location of contact: uninjured leg, upper body ipsilateral to the injured knee (Ipsi-UB), upper body contralateral to the injured knee (Contra-UB), and other [13, 15].

### Statistical analysis

All data were recorded and analyzed in Windows Microsoft Excel (Microsoft Excel 2007, Redmond, Washington, USA). We counted the number of players and calculated the percentage for the total of each variable [11]. For the results with the most cases, the binomial distribution was performed using Windows Microsoft Excel to examine the type and location of the most frequently occurring contacts.

### Ethics

#### Ethical approval

wasn’t needed because all information was publicly available. However, the athletes’ privacy was maintained as neither a direct reference to their identity nor personal data has been referred to, which would allow for their recognition, in this study [11, 12].

## Results

**Fig. 3:**
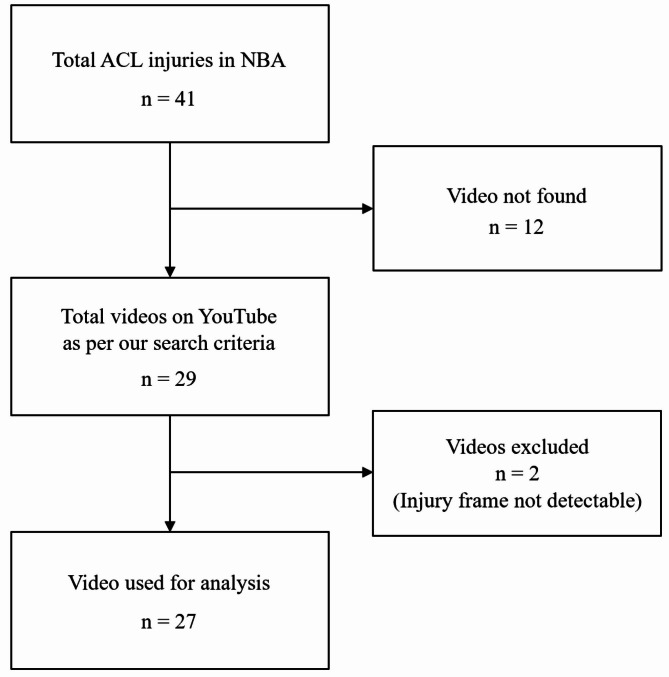
Detailed study flow-chart

The frame rate of the Youtube videos used in this paper varies from video to video, and the frame rate of Youtube videos varies from 45 to 120, depending on the case. Web research identified 41 NBA players who experienced ACL injuries from 2011 to 2021. A total of 29 videos recording ACL injuries in the NBA were found. Among them, we were able to analyze 27 videos, and we excluded two videos in which the accurate timing of ACL injury could not be clearly determined (Fig. 3).

Of the 27 cases in Tables 1, 5 were re-injured. The overall demographic data for each video are presented in Table 1.

**Table 1:** Demographic data of each video

Video	Year	Date	Age at the time of injury	Position	Medical history of ACL injuries	NBA history at the time of injury (years)	Quarter	Injured leg	Playing phase before injury
Video 01	2013	11 Feb	31	Guard	No	11	Third	Left	Offense
Video 02	2012	2 Nov	27	Guard/Forward	2007 Right	5	First	Left	Offense
Video 03	2020	27 Dec	28	Guard	2014 Jan Left	7	Third	Right	Offense
Video 04	2012	7 Jan	25	Guard	No	4	Fourth	Right	Offense
Video 05	2013	1 Apr	24	Forward	No	5	Second	Left	Offense
Video 06	2013	30 Jan	27	Guard	No	7	Not Clear	Right	Offense
Video 07	2013	19 Jan	27	Guard	No	1	Second	Right	Offense
Video 08	2017	25 Sep	22	Forward	2014 Dec Left	3	Third	Left	Offense
Video 09	2018	6 Feb	22	Forward	No	3	Second	Left	Offense
Video 10	2021	7 Jan	22	Guard	No	4	First	Left	Offense
Video 11	2017	18 Sep	20	Forward	No	0	Not Clear	Right	Rebound
Video 12	2021	14 Jun	30	Guard/Forward	No	11	Third	Right	Offense
Video 13	2021	12 Apr	24	Guard	No	5	Fourth	Left	Offense
Video 14	2021	11 Jan	24	Center	No	4	First	Left	Rebound
Video 15	2012	6 Mar	33	Guard	Left	13	Third	Left	Offense
Video 16	2015	15 Jan	23	Guard	No	4	Second	Right	Offense
Video 17	2016	16 Dec	38	Center	No	14	First	Left	Rebound
Video 18	2017	26 Jul	25	Guard	No	7	Not Clear	Left	Defense
Video 19	2019	23 Jun	29	Guard	No	9	Third	Left	Offense
Video 20	2018	7 Oct	22	Guard	No	3	Not Clear	Right	Offense
Video 21	2012	28 Apr	21	Guard	No	1	Third	Left	Offense
Video 22	2012	28 Apr	23	Guard	No	4	Fourth	Left	Offense
Video 23	2016	3 Jan	33	Guard	No	11	Third	Right	Offense
Video 24	2014	15 Dec	19	Forward	2017 Feb Left	1	Third	Left	Offense
Video 25	2017	3 Feb	21	Guard	No	3	Second	Left	Offense
Video 26	2012	9 Mar	21	Guard	No	1	Fourth	Left	Defense
Video 27	2020	2 Aug	22	Forward	No	4	Fourth	Left	Offense

Table 2 presents the classification of the 27 injured players. Among these players, 22 (81.5%) were injured in the offense phase, two in the defense phase (7.4%), and three in the rebound phase (11.1%).

**Table 2:** Classification of injuries

Categories	Number of players	Ratio (%)
Playing phase		
Offense	22	81.5
Defense	2	7.4
Rebound	3	11.1
The action performed when playing in the offense phase (n = 22)		
Dribble	3	13.6
2 steps	15	68.2
Landing	4	18.2
During which of the 2 steps that the injury occurred (n = 15)		
First step	12	80.0
Second step	3	20.0

**Fig. 4:**
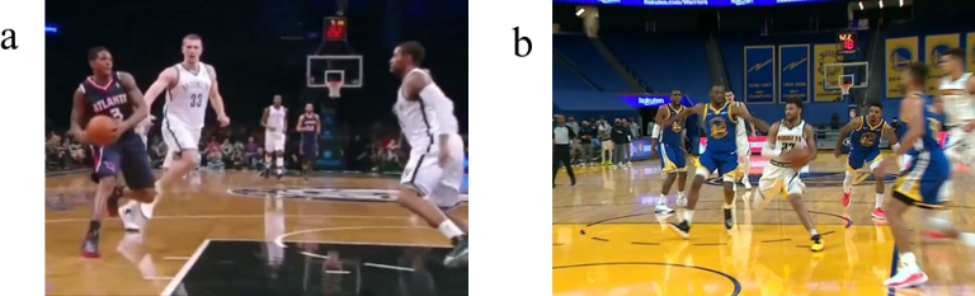
Injured player in possession of the ball in the offensive phase. These two images are of different NBA players with ACL injuries while in possession of the ball on offense. Image **(a)** shows an injured player cut in from the right. Image **(b)** shows the injured player cut in from the left

All injured players in the offense phase handled or held the ball shown in Fig. 4

**Fig. 5:**
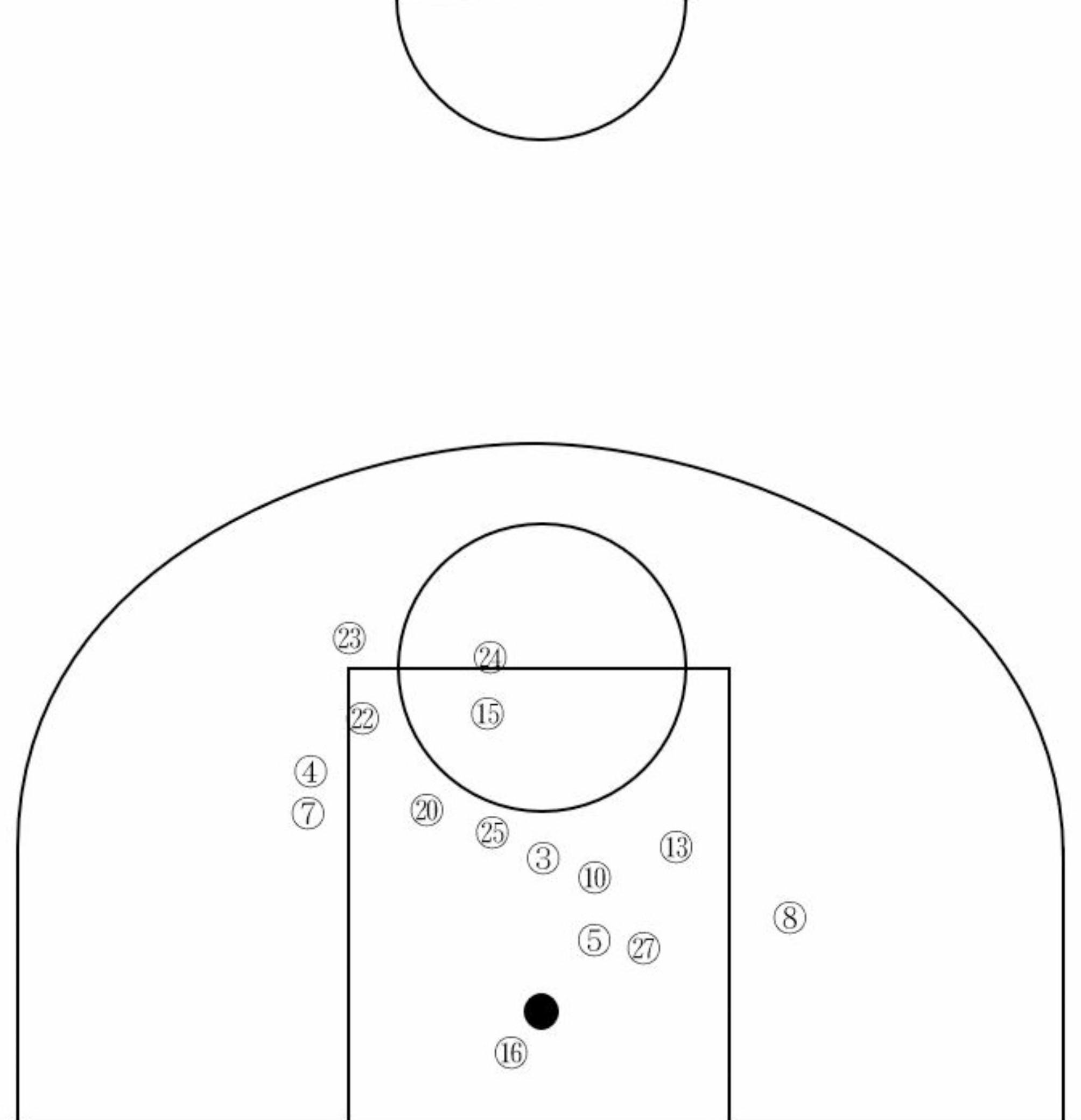
Distribution of players’ positions when injured during the 2 steps. This figure represents a scaled figure equivalent to half of a basketball court. The block dots indicate the location of the 15 players with ACL injuries during 2 steps at the time of the injury. These numbers match the number of the Video in Table 1

Of the 22 players who were injured in the offense phase, 15 (68.2%) were injured during 2 steps, three players (13.6%) during dribble, and four (18.2%) during landing. With regards to the 2 steps, 12 players (80.0%) injured their ACL during the first step and three (20.0%) during the second step. The following results were found that focused on the 15 players who were injured during the 2 steps and represented the highest number of injuries. The position of the court in which the player was injured is shown in Fig. 5.

The numbers in the figure were assigned from 1 to 15 in order of decreasing video number of the injured players during the 2 steps. All players who were injured during the 2 steps were injured inside the three-point line. The results of the detailed contact timing and type during the 2 steps are listed in Table 3.

**Table 3:** Timing and type of contact during the 2 steps and the results of the binomial distribution

At the IF-1	Number of players (%)	At the IF	Number of players
Indirect contact	11* (73.3)	Indirect contact	5
		Non-contact	6
Non-contact	4 (26.7)	Indirect contact	0
		Non-contact	4

p < 0.05 using the binomial distribution analysis

Direct contact neither existed in 1-IF nor IF

**Fig. 6:**
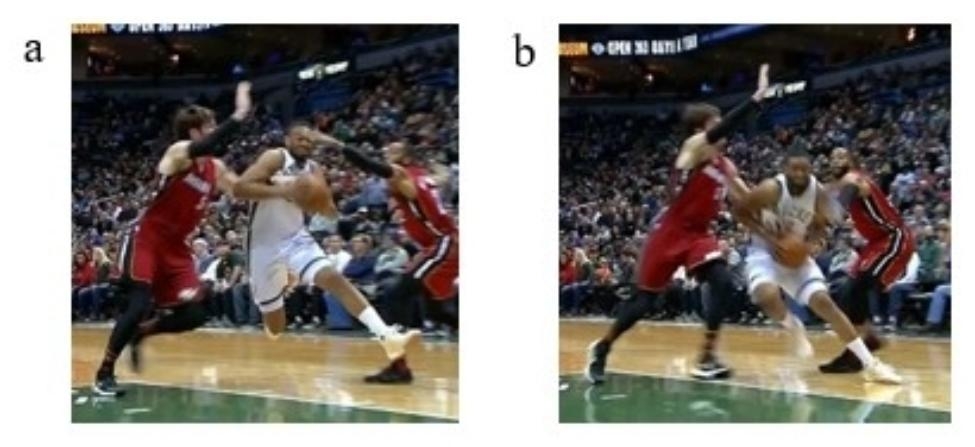
Example players who experienced Indirect contact at the IF-1 and IF during the 2 steps. These two images are from the same time series of the same NBA player. Image **(a)** shows a player who got Indirect contact at the IF-1. Image (b) shows a player who also got Indirect contact at the IF. At immediately after foot contact at the IF **(b)**, this athlete injured his ACL

**Fig. 7:**
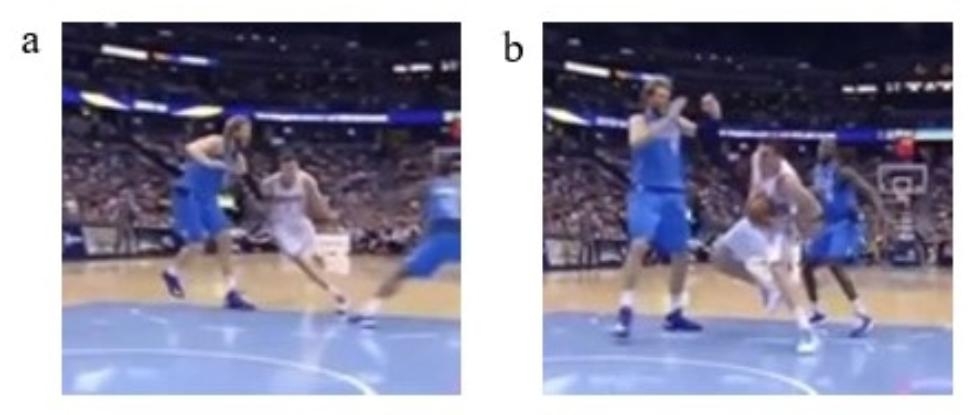
Example players with trunk leaning toward injured leg during the 2 steps. These two images are from the same time series of the same NBA player. Image **(a)** shows a player who got Indirect contact at the IF-1. Image **(b)** shows a player who did not any contact at the IF. At the IF immediately after foot contact seen in image b, this athlete injured his ACL

No player got Direct contact either one step before or at the IF; therefore, only the results of Indirect contact or Non-contact are displayed below. Eleven (73.3%) of the 15 players got Indirect contact at the IF-1, of which five continued to get Indirect contact at the IF, with six getting Non-contact. An example of the former is shown in Fig. 6 and that of the latter is shown in Fig. 7.

Of the 15 players, four (26.7%) players got Non-contact at the IF-1, and all got Non-contact at the IF. Table 4 lists the contact locations during the 2 steps.

**Table 4:** Contact location of the Indirect contact that occurred during 2 steps

	At the IF-1 n = 11	At the IF n = 5
Contra-UB	9	5
Ipsi-UB	1	0
Uninjured leg	0	0
Other	1	0

IF: Injury Frame

IF-1: one step before the injury frame

Contra-UB: upper body contralateral to the injured knee

Ipsi-UB: upper body ipsilateral to the injured knee

Of the 11 players who got Indirect contact at the IF-1, 9 (81.8%) got Indirect contact with the Contra-UB, one (9.1%) got Indirect contact with the Ipsi-UB, and the other got Indirect contact in the “other” category, as mentioned above. In addition, all five players who got Indirect contact at the IF got Indirect contact with the Contra-UB. Other than during the 2 steps, three of the four players who injured during the landing stage had landed on their leg on the goal side.Of the 15 athletes with ACL injuries during the 2 steps at the IF-1, the probability of 11 players got Indirect contact was statistically significant greater with the binomial distribution compared to 4 players got Non-contact (p = 0.042).

## Discussion

Our YouTube-based videoanalysis revealed that ACL injuries in NBA players were associated with their movements leadingup to the ACL injury. Moreover, focusing on the timing and location of the contact prior to the IF was found to be important.

In basketball, players can only perform two steps while holding the ball. During the first step, players change their direction to avoid opponents and then slow down to make a steady jump. During the second step, players jump, stop to pivot or change direction, depending on the situation. The two steps, which are considered to be relevant to ACL injuries, are characteristic movements when playing basketball, and therefore the fact that pre-injury play and contact may be mainly associated with ACL injuries falls into the category of novelty in basketball. Furthermore, it was clear that many injured players got Indirect contact with the Contra-UB before the injury.

### Playing phase and action

We found the same results as Krosshaug et al.’s previous study, in which most basketball players’ ACL injuries occurred in the offense phase. Based on our results, ACL injuries among NBA players were more frequent during the 2 steps in the offense phase, especially during the first step [9]. In addition, ACL injuries during dribbling or landing occurred in a small number of NBA players. In the following, we will discuss in detail 2 steps in which many players were injured, and also discuss dribble and landing finally.

A video analysis of ACL injuries in soccer reported that injuries were more common in complex actions involving side-step cuts after moving forward at high speed [11]. Moreover, because this action is reactive, players may lose balance by avoiding or countering their opponents and placing their lower limbs in an unsafe position [21]. The 2 steps in the present study is also reactive movement, as it often involves side-stepping or changing one’s direction diagonally to evade opponents while moving forward. Therefore, this action leads to a lower limb position in which the ACL is loaded and vulnerable to injury.

In addition, various studies have shown that changes in direction, such as side-step and cutting (which are common in two-step basketball), are more likely to cause ACL injury [22–25]. Cutting has also been reported to cause ACL injuries in basketball, with change in direction in the offense phase being performed to avoid opponents [8, 9, 26, 27]. In particular, as shown in Fig. 5, all players injured during the 2 steps were injured inside the three-point line, which is often occurs before shooting. The distance between the players was small, meaning that the need to avoid the opponent increased; thus, it can be inferred that this maneuver needs to be done dramatically and quickly. The more the players move, the more likely they are to evade their opponents. Therefore, the pivot or injured leg, is considered wide. Placing the pivot leg wide leads to a greater hip abduction angle, with the change in direction at that angle in a closed muscle chain being associated with the knee valgus and knee internal rotation moments [28].

With regards to the first step of the 2 steps, it has been reported that placing the center of pressure lateral to the body’s center of mass contributes to an increase in knee valgus moment [23, 29]. Because these moments place an increased load on the ACL, wide foot placement then becomes a risk factor for ACL injury. When players change their direction quickly to the left, right, or diagonally in front, it is assumed that the knee is not flexed sufficiently, meaning that the load placed it would increase.

A previous study found that the ground contact time is longer when the knee flexion angle is increased in the changing direction; therefore, players have a smaller knee flexion angle when they change direction quickly [30]. In fact, a video analysis comparing the joint angles between the ACL-injured and the uninjured groups reported that knee flexion former tended to be smaller than that of the latter [19]. Centrifugal contraction of the quadriceps muscle is required to resist knee flexion, which results in anterior tibial translation [13]. This may then contribute to an ACL injury.

Markolf reported that the load on the ACL increases when a knee valgus moment is applied during mild flexion [31]. Moreover, it has been reported that adopting a faster speed when changing direction to either the left or the right increases the angle of hip internal rotation, leading to knee valgus [23]. In particular, if the approach speed for a side-step change of direction is increased, it is reflected in higher ground reaction forces and higher knee abduction moments, resulting in greater knee loading [22]. From these factors, it is inferable that rapid directional changes are associated with a higher risk of ACL injury. During a quick change of direction, the speed of the first step is reported to increase the effectiveness of the attack, with the risk of ACL injury being higher during the first step than during the second step [32, 33]. This explains why more players were injured in the first step. Furthermore, these findings reveal that the 2 steps in the offense phase is an action that increases the risk of ACL injury, especially when combined with features that make it easier to evade the opponent.

Dribbling is an unstable motion that requires explosive movements and sudden changes in all directions and places excessive stress on the ACL [34]. However, it is important to move forward to go on in the game without a step limit. Therefore, compared to the 2 steps, the load on the legs is lower, and an ACL injury is less likely to occur.

Similar to other basketball studies, ACL injuries were found to occur during landing in this present research [9, 35]. From the footage, it appeared that most of the injured players were more interested in the goal or location of the ball than in their landing position during the fall after the shooting jump. In addition, in most cases, the landing foot was closer to the goal, suggesting that the lateral momentum involved in shooting affected the knee valgus reaction. Based on these findings, ACL injuries were revealed to likely occur because of the unintentional outward knee reaction force applied to the landing leg [9]. However, there are more dunk shots in the NBA than in other basketball leagues [36]. Holding the ring after dunk shots allows players to land after checking the safety of their surroundings and to slow down, meaning that cases of ACL injuries from landing after shooting in the NBA may be less frequent.

### Contact with opponent

In this study, it was found that ACL injuries occurred during the 2 steps, either with Indirect contact or without any contact. Indirect contact was observed not only at the IF but also at the IF-1, with many players getting Indirect contact with the Contra-UB. In addition, when looking at players who got Indirect contact at the IF-1, the number of players who also got Indirect contact at the IF and those who did not were only slightly different. This suggests that Indirect contact at the IF-1 is important for understanding ACL injuries.

Our results reveal that many ACL injuries involve Indirect contact with the Contra-UB. Considering that almost all injured players were in the offense phase, they then had to handle or hold the ball on the opposite side of the opponents, with their upper body turning toward the ball side to prevent their opponent from stealing it. Thus, it was possible that their hip and trunk rotated to the opposite side of the opponent; namely, to the injured leg side. To counter the Indirect contact while maintaining this state, players may have taken their pivot leg wide and performed knee extension, hip adduction, and hip internal rotation. As mentioned above, the posture wherein the pivot leg is placed widely and a decrease in the knee flexion angle occurs increases the risk of ACL injury. Previous studies have reported that the combination of trunk rotation to the injured leg and hip adduction is a strong predictor of increases to the knee valgus moment, which is associated with ACL loading [31, 37]. Therefore, Indirect contact with the Contra-UB may lead to the adaptation postures and movements that have a high risk of ACL injuries.

The reasons for players who were injured got Indirect contact all the time from one step before to at the IF may be related to the aforementioned observations. On the other hand, as shown in Fig. 6, players who got Indirect contact at the IF-1 but not at the IF may have landed on the injured foot with the upper body in an abnormal position [13]. Because there was Indirect contact with the Contra-UB, the trunk would have been leaning toward the injured side. It has been reported that the knee valgus moment is larger when the trunk is leaned to the planted leg side in comparison to when it is leaned to the unplanted leg side during the side-step (28). Moreover, the more the trunk leans toward the planted leg side, the more the ground reaction force passes outside the knee, and the greater the knee abduction moment, which is a known risk factor for ACL injury [22, 38, 39]. Focusing on the hip joint, trunk inclination also increases the external hip abduction moment and, in order to counter this, hip adduction is further applied to stabilize the trunk and pelvis [40]. These mechanisms may then contribute to the knee valgus moment, which can then cause an ACL injury. Impaired lateral control of the trunk also correlates with ACL incidence, with unexpected contact in sports often occurring, which then result in a of control [41]. From these discussions, we can conclude that Indirect contact is likely a risk factor for ACL injury if there is Non-contact at the time of injury but there is Indirect contact prior to it. This study suggests that it is important to consider Indirect contact before injury when considering ACL injuries in basketball players.

Some limitations of this study be noted. First, we did not include controls for players who performed actions without receiving an injury. Therefore, a comparison between injured and non-injured players remains explored. Based on the results of the present study, which found that ACL injuries are more common during the 2 steps among NBA players, it is necessary to analyze whether there is a difference between injured and uninjured players during 2 steps in future studies. Second, we could not examine, from a video analysis alone, how much Indirect contact affects players’ play and movements. The video data from this study does not provide biological data, so it is not possible to provide biological evidence of risk factors or mechanisms of injury. As such, conducting a detailed analysis of Indirect contact and Non-contact injuries in future studies is important. The third limitation is the analysis accuracy, which was limited due to the camera angles and the quality of the YouTube videos. Although it was possible to determine the movement, presence/absence, and location of the contact, it was difficult to measure the joint angle and length. This is because that the video angle was not always obtained from the plane of motion, with the scale of the videos not being uniform. YouTube videos are simply a tool for capturing the action, situation, and moment of injury. The fourth limitation is the number of datasets, in which the scale of the dataset seems small, so this result may reflect only some of the NBA’s trends.

As the analysis could be further developed by incorporating deep learning methods, the study design is such that deep learning methods can be used for similar studies in the future. In this study, two physiotherapists reached a consensus; on the other hand, deep learning may make more accurate and objective assessments than humans by learning from correct data. In addition, we could use deep learning to extract new features that humans do not well capture and to analyze the movements and postures that lead to ACL injuries.

## Conclusions

Through a YouTube-based video analysis, we found that ACL injuries in NBA players mainly occur during the 2 steps, the characteristic action of basketball with high risk of ACL injury. It was also revealed that many of the injuries occurred during the first step of the 2 steps inside the three-point line, in which the speed and scale of the action were particularly important. Furthermore, Indirect contact with the upper body before injury associated with an increased risk of ACL injury.

## Data Availability

The datasets used and/or analyzed during the current study are available from the corresponding author on reasonable request.

## Abbreviations

ACL: Anterior Cruciate Ligament
NBA: National Basketball Association
IF: Injury Frame
IF-1: One step before the injury frame
Direct contact: Contact with the injured leg
Indirect contact: Contact to an area other than the knee
Non-contact: No contact with other players
Contra-UB: Upper body contralateral to the injured knee
Ipsi-UB: Upper body ipsilateral to the injured knee
